# Integrating AI in Healthcare Management Education

**DOI:** 10.1093/geroni/igaf122.1550

**Published:** 2025-12-31

**Authors:** Frances Hawes

**Affiliations:** University of Wisconsin-Eau Claire, University of Wisconsin-Eau Claire, Wisconsin, United States

## Abstract

This study explores AI’s role in healthcare education, focusing on nursing home regulatory citations. The study aimed to assess students’ ability to interpret F-tag citations, apply them in quality improvement strategies, and distinguish between AI-generated data and manual findings. Students first researched assigned F-tags using the CMS State Operations Manual. In the second phase, they used AI to retrieve and analyze citation information, adjusting queries to evaluate AI output variations. Learning outcomes were assessed through two completed worksheets (n = 32), class discussions, and student reflections on AI’s role in understanding citations and regulatory management. Results indicate that AI simplifies complex regulations, making them more accessible and helping students identify gaps in their initial understanding. Students found AI useful for citation management, recognizing its potential to automate analysis, identify trends, and assist in corrective action planning. AI was also seen as a valuable tool for compliance monitoring and training, providing real-time updates and tracking regulatory changes. However, concerns emerged regarding AI’s limitations, including inaccurate responses, the risk of over-reliance, and the need for human oversight to ensure accuracy and ethical use. Ethical discussions particularly focused on AI-generated corrective action plans and the importance of maintaining human responsibility in regulatory decision-making. This study highlights AI’s potential in healthcare education and its broader applications in policy analysis and patient safety.

